# The Impact of Comprehensive Rehabilitation on the Exercise Capacity of Patients after COVID-19

**DOI:** 10.3390/arm91060037

**Published:** 2023-11-14

**Authors:** Alicja Mińko, Agnieszka Turoń-Skrzypińska, Aleksandra Rył, Iwona Rotter

**Affiliations:** Department and Unit of Medical Rehabilitation and Clinical Physiotherapy, Pomeranian Medical University, 71-210 Szczecin, Poland; agi.skrzypinska@gmail.com (A.T.-S.); aleksandra.ryl@pum.edu.pl (A.R.); iwrot@wp.pl (I.R.)

**Keywords:** COVID-19, rehabilitation, 6-minute walk test

## Abstract

**Highlights:**

**What are the main findings?**

- The comprehensive post-COVID-19 rehabilitation program is an effective intervention that improves the results of the 6-minute walk test.
- Comprehensive rehabilitation after COVID-19 treatment in stationary conditions increases physical capacity.

**What is the implication of the main finding?**

- Patients after COVID-19 treatment can benefit from comprehensive rehabilitation.
- Comprehensive rehabilitation in patients after COVID-19 treatment reduces perceived fatigue after exercise.

**Abstract:**

Coronavirus Disease 2019 (COVID-19) is a complex disease that affects multiple body systems, including the respiratory, cardiovascular, neurological, and muscular systems. It is estimated that approximately half of the patients after the treatment for COVID-19 experience persistent symptoms that lead to a decreased physical capacity. Scientific recommendations suggest that cardiovascular and respiratory rehabilitation programs should be implemented in patients who have completed treatment for COVID-19. Therefore, the objective of this study was to evaluate the impact of comprehensive rehabilitation on the exercise capacity of patients after COVID-19 treatment. The study included 146 patients after the treatment for COVID-19 who were eligible for therapeutic rehabilitation. The exercise capacity was assessed using the 6-minute walk test (6MWT). The results showed that patients who underwent rehabilitation had an average increase of 23.83% in their 6MWT score compared to the baseline. A comprehensive rehabilitation program including breathing exercises, aerobic training, and strength and endurance exercises is an effective intervention that can improve the physical capacity of patients after COVID-19 treatment.

## 1. Introduction

COVID-19 is a complex disease caused by the severe acute respiratory syndrome coronavirus 2 (SARS-CoV-2) virus that affects multiple systems in the body, including the respiratory, cardiovascular, neurological, and muscular systems. Persistent symptoms, such as shortness of breath, chest pain/discomfort, and fatigue, are reported by about half of the patients after COVID-19 treatment, even 2–3 months after the infection [1,2]. These symptoms are associated with a decreased physical capacity, which can lead to avoidance of physical activity, deterioration of the quality of life, and ultimately an inability to perform basic activities of daily living [3,4]. The primary mechanism of persistent symptoms after COVID-19 may be due to dysfunction of the coronary vessels, resulting in a decrease in the maximal oxygen uptake threshold, a measure of physical capacity [5,6,7].

6MWT is a widely used clinical tool for assessing exercise capacity and is often utilized as a predictor of mortality risk in patients with lung diseases [8]. Recently, it has also been applied to patients with COVID-19. Research indicates that patients with COVID-19 who cover a shorter distance in the 6MWT are at a higher risk of desaturation [9]. Studies also show a beneficial effect of comprehensive rehabilitation on the distance covered in the 6MWT in patients after COVID-19 [5,10,11].

Individuals with COVID-19 require individualized rehabilitation based on their specific needs. According to recommendations, rehabilitation procedures can be carried out in different settings, including inpatient, outpatient, or home-based care. The primary goal of rehabilitation after COVID-19 is to alleviate shortness of breath, improve overall fitness, and increase physical capacity [12,13,14].

Comprehensive rehabilitation is a fundamental approach for managing individuals with chronic lung diseases [12]. This approach involves personalized rehabilitation programs that include aerobic exercises, breathing exercises, and muscle-strengthening exercises. According to scientific recommendations, these programs should also be implemented in patients after COVID-9-19 treatment [15,16].

The purpose of this study was to evaluate the impact of comprehensive rehabilitation on exercise capacity in patients after COVID-19. The effectiveness of rehabilitation was measured using the 6MWT, which measures the distance covered, with the minimum clinically important difference of 30 m for the 6MWT used as an additional goal [17]. The study also aimed to determine the association between the difference in the 6MWT distance before and after rehabilitation and factors such as sex, age, body mass index, comorbidities, the presence of pneumonia during SARS-CoV-2 infection, the time after which rehabilitation was started and the duration rehabilitation and hospitalization.

## 2. Materials and Methods

The study was conducted from 31 May 2021 to 30 September 2022 at St. Charles Borromeo Rehabilitation Hospital in Szczecin, Poland. The study involved 171 participants staying at the Post-COVID-19 Rehabilitation Department, where medical rehabilitation of patients after SARS-CoV-2 infection was carried out in stationary conditions.

Each patient gave written informed consent to participate in this study and to use data from their medical records. Every effort has been made to protect the privacy and anonymity of patients. The study was conducted in accordance with the current version of the Declaration of Helsinki. Approval to conduct the study was obtained from the Bioethics Committee of the Pomeranian Medical University in Szczecin (decision no. KB-0012/15/2021).

### 2.1. Characteristics of the Study Group

The qualification for the post-COVID-19 rehabilitation program in stationary conditions was based on the guidelines of the National Health Fund in Poland [18]. The qualification for rehabilitation was carried out by a doctor specializing in medical rehabilitation. Patients with post-COVID-19 complications were qualified for rehabilitation, which was assessed on the basis of the Post-COVID-19 Functional Status (PCFS) Scale (score 1–4), the Medical Research Council (score < 5), and the modified Medical Research Council (score ≥ 1). The Post-COVID-19 Functional Status Scale is a five-point scale used to identify patients with functional limitations related to many aspects of health after COVID-19 [19]. The Medical Research Council is a scale used to test muscle strength. The score ranges from 0 to 5, where 0 is no muscle tone and 5 is normal muscle strength [20]. The modified Medical Research Council is a five-point scale assessing the severity of dyspnea. A score of 0 indicates shortness of breath only during strenuous exercise, while a score of 4 indicates shortness of breath that prevents leaving the house [21].

Other inclusion criteria were age > 18 years, confirmed COVID-19 diagnosis by a positive polymerase chain reaction test for SARS-CoV-2, and a period no longer than 12 months after the end of COVID-19 treatment. The end of COVID-19 treatment was defined as the date of the end of home isolation, discharge from the hospital, or isolation center. The diagnostic test required for qualification for rehabilitation was also a chest X-ray with a description, performed after the completion of the treatment in the acute phase of the disease.

The exclusion criteria for participation in this study were age < 18 years, refusal to participate in the study, interrupted 6MWT (due to significant dyspnea, fatigue, balance disorders, or fainting), contraindications to 6MWT (symptoms of unstable angina or myocardial infarction in the last month, resting heart rate ≥ 120 beats per minute, systolic blood pressure ≥ 180 mmHg, diastolic blood pressure ≥ 100 mmHg). Patients with musculoskeletal disorders preventing the independent completion of the 6MWT were also excluded from the study.

**Figure 1:**
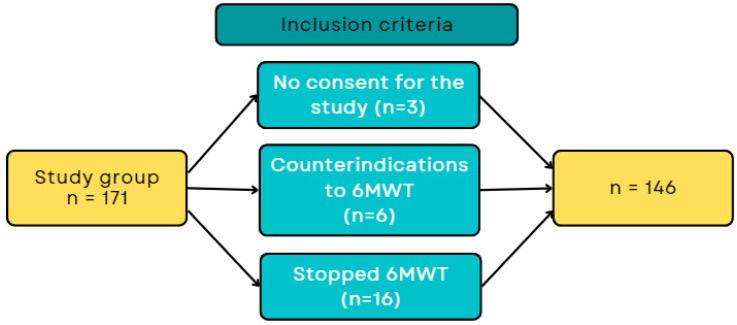
The flowchart of patient qualification for the study.

In total, 146 patients were included in the study, taking into account all inclusion and exclusion criteria (Figure 1).

### 2.2. Rehabilitation Procedure

**Figure 2:**
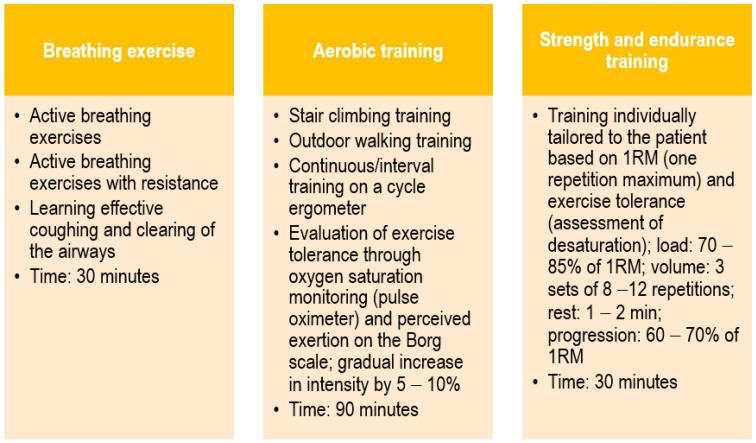
The rehabilitation procedure.

The examined patients participated in a comprehensive rehabilitation program after COVID-19. The comprehensive rehabilitation program included breathing exercises, aerobic training, and strength and endurance training. The detailed procedure is presented in Figure 2. Rehabilitation activities were conducted six times a week, from Monday to Saturday. Throughout the entire rehabilitation period, the patient was under medical, nursing, and physio-therapeutic care. The minimum rehabilitation time that the patient had to undergo was 2 weeks. The decision to extend rehabilitation (up to a maximum of 6 weeks) was made by the attending physician based on a comparison of the current examination and test results with those carried out before the start of rehabilitation. This includes an exercise test (6-minute walk test) with an assessment of exercise tolerance, an assessment of the severity of shortness of breath (on the mMRC scale), and a spirometric assessment of the functional function of the respiratory system.

The exercise capacity was assessed based on the 6MWT on admission and at discharge from the rehabilitation ward. The 6MWT was conducted according to the American Thoracic and European Respiratory Society standards [17]. The 6MWT was performed along a straight, hard-surfaced corridor measuring 30 m, marked with cones at both ends. The distance covered by the patient in 6 minute was measured. The predicted 6MWD was calculated using the formulas:

- 6MWT [meter] = (7.57 × height [centimeter]) − (5.02 × age [years]) − (1.76 × weight [kilogram]) − 309 (for men)
- 6MWT [meter] = (2.11 × height [centimeter]) − (2.29 × weight [kilogram]) − (5.78 × age [years]) + 667 (for women).

Results were expressed as an absolute value and as a percentage of predicted normal values for each patient. Based on the obtained data and the formula: velocity = distance/time (meter/second), the average speed at which the distance was covered was calculated. Before and after the 6MWT, measurements of oxygen saturation (%), heart rate and systolic and diastolic blood pressure (mmHg) were taken. The degree of fatigue was measured at the end of the 6MWT using the Borg Scale. The scale uses responses ranging from 6 to 20. Lower numbers on the Borg Scale indicate no fatigue, and 20 indicates maximum fatigue [22].

At the beginning and end of rehabilitation, a spirometry test was also performed to assess lung function [23]. The severity of dyspnea was assessed using the mMRC scale.

On admission to the rehabilitation ward, a demographic interview was conducted to obtain patient data. Information regarding the course of the disease and treatment, as well as coexisting conditions, was obtained from medical records.

### 2.3. Statistical Methods

Statistical analysis was performed using Statistica 13.1 software (StatSoft, Inc., Tulsa, OK, USA). Descriptive statistics including the number of patients, patient percentages, mean, and standard deviation were used to characterize the study group. The normality of distribution was assessed using the Shapiro–Wilk test. Student’s *t*-test and Mann–Whitney U-test were used to analyze differences between two groups, while the Kruskal–Wallis test or ANOVA test was used to analyze differences between multiple groups. The correlation analysis was performed using Spearman’s Rho test. The chi-squared test was used to test nominal variables. The *t*-test for dependent samples and the Wilcoxon test were used to test dependent variables. A multivariable logistic regression analysis was performed with the minimum clinically important difference of 30 m for chronic respiratory diseases as the predictor of improvement in the 6MWT distance. The model was adjusted for gender, age, body mass index, pneumonia, and length of hospitalization. A statistical significance was attributed to results where the p-value was lower than 0.05.

## 3. Results

The mean age in the study group was 64.1. The mean height and weight of the study population were 168.42 and 82.7, respectively. The mean body mass index was 29.04. Detailed information regarding the characteristics of the study group is presented in Table 1.

Table 2 presents the results of the 6MWT parameters before and after rehabilitation. 

After rehabilitation, patients achieved a longer 6MWT distance by an average of 23.83%. The walking speed also increased. After rehabilitation, the resting systolic and diastolic blood pressure decreased. After rehabilitation, patients had better saturation both at rest and after exercise. After rehabilitation, the level of fatigue measured by the Borg scale and the severity of shortness of breath measured by the Modified Medical Research Council scale decreased.

Table 3 shows the relationships between groups and 6MWT results before and after rehabilitation. After rehabilitation, the men achieved a better 6MWT result. People over 65 years of age achieved lower 6MWT results both before and after rehabilitation. People with a BMI over 30 achieved lower 6MWT results after rehabilitation. Patients with diabetes and hypertension had worse 6MWT results both before and after rehabilitation.

Table 4 shows correlations between age, degree of obesity, length of hospitalization in COVID-19, length of rehabilitation, time after which rehabilitation began, and the difference in the 6MWT distance before and after rehabilitation. As age and BMI increased, the difference in the 6MWT distance before and after rehabilitation decreased.

The results of the multivariate regression analysis are presented in Table 5. The analysis did not reveal statistically significant correlations between examined variables.

## 4. Discussion

In this study, we evaluated a group of patients who, after completing COVID-19 treatment, participated in comprehensive rehabilitation in stationary conditions, based on breathing exercises, aerobic training, and strength and endurance exercises. Our study shows that a supervised exercise program lasting 2 to 6 weeks significantly improves health indicators of patients’ physical performance. There was a significant improvement in 6MWT results by an average of 120 m. The degree of perceived fatigue after exercise decreased by an average of 1.87 points, as did the severity of shortness of breath (a decrease of 1.82 points). The average post-exercise SpO2 increased by an average of 1.55%. Our previous studies [23] conducted on the same group of patients also demonstrate significant improvement in functional lung function. The spirometry test showed that after rehabilitation patients showed a significant improvement in parameters such as forced vital capacity (FVC), forced expiratory volume in the first second (FEV1), peak expiratory flow (PEF), maximal mid-expiratory flow (MMEF), maximal expiratory flow 75% (MEF75), and maximum expiratory flow 50% (MEF50). 

Liu et al. [24] during a 6-week pulmonary rehabilitation program noted an improvement both in the 6MWT (from 162.7 m to 212.3 m) and in the results of spirometric tests—an increase in FVC (1.79 L before to 2.36 L after) and DLCO (60.3% before to 78.1% after). Łoboda et al. [25] during a 3-week comprehensive rehabilitation program, including interval training on a bicycle ergometer, general kinesitherapy, and breathing exercises, noted an improvement in exercise capacity by an average of 42.5 m in 6MWT, as well as a reduction in shortness of breath during daily activity (ΔmMRC, −1 point). However, no significant changes were found in spirometric parameters. Only an average improvement of 1.92% in PEF was observed. In turn, Hermann et al. [26] noted a significant improvement in 6-MWT results (an increase of 130 m) during a 2–4 week rehabilitation program including aerobic exercises and strength training. Similar results are presented by Hockele et al. [27], who used a comprehensive rehabilitation model consisting of inspiratory muscle training, aerobic exercises, and peripheral muscle strength exercises. The authors observed an improvement in functional capacity, confirmed by a 6MWT, along with an increase in the distance covered by an average of 119.1 m. An improvement in lung function was also demonstrated, confirmed by a spirometry test (FVC and FEV1).

Observations from other studies show that pulmonary rehabilitation plays a key role in improving lung function and overall physical fitness of patients after COVID-19. Pulmonary rehabilitation is generally recommended as the primary rehabilitation strategy for patients with persistent respiratory symptoms. Additionally, it is worth noting that supervised and individually adapted low-to-moderate intensity training, including both resistance and endurance exercises, has been shown to be an effective, safe, and well-tolerated form of rehabilitation intervention in cases of recovery from COVID-19 [12,28].

The indicated studies show that the duration of rehabilitation after COVID-19 ranged from 2 to 6 weeks. Physical performance improved with an increasing 6MWT distance in each of these examples. Interestingly, Hermann et al. [26] reported identical improvements in the 6MWT in previously mechanically ventilated patients as in unventilated patients and with no significant differences in patient characteristics. Gloeckl et al. [29] assessed the effectiveness of rehabilitation after COVID-19 depending on the course of the acute phase. They noted a similar improvement in physical capacity in patients with mild/moderate COVID-19 and in patients after the severe/critical acute phase. Similarly, in our analysis, the time of rehabilitation initiation and the occurrence of pneumonia had no impact on the effectiveness of rehabilitation. This suggests the need to conduct further analyses and comparisons with other patient groups, which could contribute to a more comprehensive understanding of the impact of rehabilitation on recovery after COVID-19.

Our study did not include a control group. However, Carvalho et al. [30] showed that both the study group undergoing rehabilitation and the control group without rehabilitation improved 6MWT results. However, the study group showed significantly better results compared to the control group. The results suggest that physical fitness may spontaneously improve over time. However, a comprehensive post-COVID-19 rehabilitation program may contribute to faster recovery from COVID-19.

The effectiveness of rehabilitation may also depend on other factors such as age, gender, or comorbidities. Identifying potential risk factors associated with poorer recovery from COVID-19 is important because identifying those at higher risk can help inform health care planning for these patients [31]. Many publications indicate that the female gender is significantly associated with a greater risk of a more severe course of COVID-19 [32,33,34]. However, research conducted by Łoboda et al. [25] shows that women recorded better results in the 6MWT study after rehabilitation. Our research shows a similar relationship. In men after rehabilitation, the average 6MWT was 515.54 m, and in women 469.2 m, which is 91.37% pred and 101.44% pred, respectively. There is evidence that older age may be associated with long-term symptoms of COVID-19 [35,36]. Our research proves that as the age of rehabilitated patients increased, the effectiveness of rehabilitation expressed in the difference in the distance of 6MWT before and after rehabilitation decreased.

According to various reports published around the world, infection and mortality due to COVID-19 were more common in people suffering from chronic diseases such as diabetes and obesity [31,37]. Our research shows that patients with diabetes, hypertension, and obesity also have worse 6 MWT results in response to comprehensive rehabilitation. There is a need for further research to better understand the long-term effects of rehabilitation and to develop optimal therapeutic protocols for different patient groups. Different patients have different degrees of dysfunction, so personalized physiotherapy plans should be developed taking into account the age, gender, lifestyle, comorbidities, and physical conditions of the patients [15].

### Study Limitations

The limitation of our study was the different times of starting rehabilitation, but it was not longer than 12 months after the recovery from COVID-19. The duration of the rehabilitation cycle was also not uniform, ranging from 2 to 6 weeks, which may have affected the final results. Another limitation of our study is that we do not know whether any of the patients were taking steroids and/or neuromuscular blocking drugs, which could have influenced the final results. The study did not include radiological and tomographic assessment lungs of rehabilitated patients. The strength of the study was the supervised rehabilitation process, with all patients performing exercises at the rehabilitation ward under the constant supervision of physiotherapists. Future studies should investigate physical fitness using more precise measurements such as cardiopulmonary exercise testing.

## 5. Conclusions

A comprehensive rehabilitation program based on breathing exercises, aerobic training, and strength and endurance exercises is an effective intervention that can improve the physical capacity of patients after COVID-19 treatment. During rehabilitation, special attention should be paid to the elderly, as well as people with coexisting diabetes, hypertension, and obesity.

## Figures and Tables

**Table 1:** Characteristics of the study group.

Variable		*n*	%
Gender	Female	79	54
	Male	67	46
Age	30–45 years	15	10
	46–60 years	27	18
	61–75 years	82	56
	76–90 years	22	15
Nutritional status (BMI)	18.5–24.99 (norm)	29	20
	25.0–29.9 (overweight)	56	38
	30.0–34.99 (1st degree obesity)	43	29
	35.0–39.99 (2nd degree obesity)	14	10
	over 40 (3rd degree obesity)	4	3
Hospitalization	Yes	101	69
	No	39	27
Length of hospitalization	1–5 days	5	3
	6–10 days	10	7
	11–15 days	26	18
	16–20 days	16	11
	More than 20 days	44	30
Pneumonia during COVID-19 infection	Yes	107	73
	No	33	22
Mechanical ventilation	Yes	12	8
	No	134	92
Oxygen therapy during hospitalization	Yes	95	65
	No	51	35
The duration of rehabilitation	2–3 weeks	7	5
	3–4 weeks	60	41
	4–5 weeks	45	31
	5–6 weeks	34	23
Comorbidities	Diabetes	34	23
	Hypertension	79	54
	Asthma	16	11
	COPD	7	5
Smoking status	Yes	16	11
	No	130	89

Legend: *n*—number, BMI—body mass index, COPD—chronic obstructive pulmonary disease.

**Table 2:** Relationships between 6MWT parameters before and after rehabilitation.

Variable		Before Rehabilitation (*n* = 146)	After Rehabilitation (*n* = 146)	*p*
		M (SD)	M (SD)	
6MWT distance (m)		370.03 (122.63)	490.47 (144.26)	<0.001 *
6MWT distance (%predicted)		74.61 (23.53)	98.44 (24.89)	<0.001 *
6MWT speed (m/s)		1.03 (0.34)	1.36 (0.4)	<0.001 *
HR (bpm)	Rest	78.25 (15.45)	77.27 (14.34)	0.815
	End	88.15 (18.01)	96.64 (20.25)	<0.001 *
SBP (mmHg)	Rest	128.77 (18.46)	125.41 (14.9)	0.040 *
	End	136.1 (21.08)	142.6 (21.03)	<0.001 *
DBP (mmHg)	Rest	79.41 (11.43)	77.31 (9.62)	0.023 *
	End	81.64 (11.67)	82.14 (11.53)	0.888
SpO2 (%)	Rest	94.93 (2.94)	95.86 (2.18)	<0.001 *
	End	94.18 (4.52)	95.73 (3.02)	<0.001 *
Borg’s scale		12.26 (2.28)	10.39 (2.61)	<0.001 *
mMRC		2.55 (0.66)	0.73 (0.72)	<0.001 *

Legend: 6MWT—6-minute walk test; HR—heart rate; SBP—systolic blood pressure; DBP—diastolic blood pressure; SpO2—oxyhemoglobin saturation; Rest—at rest before the 6MWT; End—at the end of the 6MWT; mMRC—Modified Medical Research Council scale; s—second; m—meter; bpm—beats per minute; M—mean; SD—standard deviation; n—number of patients; *p*—statistical significance; * *p* < 0.05.

**Table 3:** Comparison of distances in the 6MWT before and after rehabilitation between groups.

Variable	Before Rehabilitation		*p*	After Rehabilitation	*p*
	M (SD)			M (SD)	
Sex	Woman	351.43 (109.03)	0.079	469.2 (122.28)	0.027 *
	Man	391.97 (134.49)		515.54 (163.93)	
Pneumonia	Yes	366.27 (124.02)	0.459	488.69 (143.15)	0.490
	No	387.3 (115.41)		505.06 (152.48)	
BMI	<25	385.43 (124.11)	0.108	510.8 (151.65)	0.003 *
	25–30	384.91 (123.94)		515.13 (141.51)	
	>30	348.5 (118.34)		434.84 (137.76)	
Age	<65	422.18 (107.04)	<0.001 *	573.62 (115.44)	<0.001 *
	>65	322.61 (119.92)		430.78 (133.34)	
Diabetes	Yes	327.71 (123.83)	0.005 *	412.94 (146.11)	<0.001 *
	No	388.77 (115.54)		510.29 (138.25)	
Hypertension	Yes	355.09 (121.35)	0.021 *	448.21 (142.32)	<0.001 *
	No	405.13 (113.68)		555.36 (130.49)	
Asthma	Yes	342.88 (152.08)	0.316	441.89 (167.23)	0.214
	No	374.59 (116.71)		486.81 (144.40)	
Smoking status	Yes	368.56 (94.03)	0.923	458.81 (142.23)	0.501
	No	371.7 (124.93)		485.06 (148.26)	

Legend: 6MWT—6-minute walk test; BMI—body mass index; M—mean; SD—standard deviation; *p*—statistical significance; * *p* < 0.05.

**Table 4:** Correlations between age, obesity level, length of hospitalization in COVID-19, length of rehabilitation, time after which rehabilitation began, and the difference in the 6MWT distance before and after rehabilitation.

Pair of Variables		R	*p*
Difference in the 6MWT distance before and after rehabilitation (m)	Age	−0.31984	<0.001 *
	BMI	−0.26962	<0.001 *
	Length of hospitalization	0.12359	0.218
	Length of rehabilitation	0.04552	0.589
	Time (months) after which rehabilitation began	−0.16260	0.052

Legend: 6MWT—6-minute walk test; BMI—body mass index; R—correlation coefficient; *p*—statistical significance; * *p*-value < 0.05.

**Table 5:** Multivariate logistic regression model predicting the 6MWT improvement.

Outcome		OR (95% CI)	*p*
Gender	Male	1.0 (0.996–1.004)	0.906
Age	>60 years	1.004 (0.999–1.008)	0.093
Nutritional status (BMI)	Norm	1.073 (0.565–2.037)	0.829
	overweight	0.998 (0.994–1.003)	0.470
	1st-degree obesity	1.0 (0.996–1.004)	0.978
	2nd-degree obesity	0.98 (0.705–1.363)	0.904
	3rd-degree obesity	0.91 (0.67–1.298)	0.467
Hospitalization		1.001 (0.995–1.007)	0.727
Pneumonia in the course of COVID-19		0.998 (0.991–1.005)	0.538

Legend: BMI—body mass index; *p*—statistical significance, OR—odds ratio, CI—confidence interval.

## Data Availability

The data that support the findings of this study are available from the corresponding author, (A.M.), upon reasonable request.
